# Misdiagnosis of chronic pulmonary aspergillosis as pulmonary tuberculosis at a tertiary care center in Uganda: a case series

**DOI:** 10.1186/s13256-021-02721-9

**Published:** 2021-03-30

**Authors:** Richard Kwizera, Andrew Katende, Felix Bongomin, Lydia Nakiyingi, Bruce J. Kirenga

**Affiliations:** 1 Department of Research, Infectious Diseases Institute, College of Health Sciences, Makerere University, P.O.BOX, 22418 Kampala, Uganda; 2 Makerere University Lung Institute, College of Health Sciences, Makerere University, Kampala, Uganda; 3 Department of Medicine, Makerere University College of Health Sciences, Makerere University, Kampala, Uganda; 4 Department of Medical Microbiology & Immunology, Faculty of Medicine, Gulu University, Gulu, Uganda

**Keywords:** Chronic pulmonary aspergillosis, Lateral flow device, *Aspergillus*-specific IgG, Diagnosis, Resource-limited settings, Case report

## Abstract

**Background:**

Diagnosis of chronic pulmonary aspergillosis (CPA) is based on a combination of clinical symptomatology, compatible chest imaging findings, evidence of *Aspergillus* infection and exclusion of alternative diagnosis, all occurring for more than 3 months. Recently, a rapid, highly sensitive and specific point-of-care lateral flow device (LFD) has been introduced for the detection of *Aspergillus*-specific immunoglobulin (Ig)G, especially in resource-limited settings where CPA is underdiagnosed and often misdiagnosed as smear-negative pulmonary tuberculosis (PTB). Therefore, in our setting, where tuberculosis (TB) is endemic, exclusion of PTB is an important first step to the diagnosis of CPA. We used the recently published CPA diagnostic criteria for resource-limited settings to identify patients with CPA in our center.

**Case presentation:**

Three Ugandan women (45/human immunodeficiency virus (HIV) negative, 53/HIV infected and 18/HIV negative), with a longstanding history of cough, chest pain, weight loss and constitutional symptoms, were clinically and radiologically diagnosed with PTB and empirically treated with an anti-tuberculous regimen despite negative microbiological tests. Repeat sputum Mycobacteria GeneXpert assays were negative for all three patients. On further evaluation, all three patients met the CPA diagnostic criteria with demonstrable thick-walled cavities and fungal balls (aspergilomas) on chest imaging and positive *Aspergillus*-specific IgG/IgM antibody tests. After CPA diagnosis, anti-TB drugs were safely discontinued for all patients, and they were initiated on capsules of itraconazole 200 mg twice daily with good treatment outcomes.

**Conclusions:**

The availability of simple clinical diagnostic criteria for CPA and a LFD have the potential to reduce misdiagnosis of CPA and in turn improve treatment outcomes in resource-limited settings.

**Supplementary Information:**

The online version contains supplementary material available at 10.1186/s13256-021-02721-9.

## Background

Tuberculosis (TB) is one of the most common causes of death and the leading cause of death from a single infectious agent worldwide. The highest burden is found in Africa and Asia mostly linked to the human immunodeficiency virus (HIV) epidemic in these regions [1]. The 2020 World Health Organization (WHO) report on TB showed that globally 10 million people developed TB in 2019, with an estimated 1.2 million deaths among HIV-negative people and an additional 208,000 deaths from TB among HIV-positive people [2]. Due to residual lung damage, pulmonary tuberculosis (PTB) is an important risk factor for other chronic respiratory diseases including chronic obstructive pulmonary disease (COPD), bronchiectasis and aspergillosis [3].

Chronic pulmonary aspergillosis (CPA) is a slowly progressive and destructive parenchymal lung disease mostly caused by *Aspergillus fumigatus* [4] and affecting both immunocompetent and immunocompromised patients commonly with previous or underlying lung diseases [5]. CPA affects an estimated 3 million people worldwide [6, 7] and 62,000 people in Uganda [8]. More than half of cases of CPA occur as a complication of treated PTB; CPA and PTB can co-exist, posing a challenge in distinguishing the two clinically and on radiology [9]. This group of patients is often misdiagnosed and managed as smear-negative PTB [10, 11]. With the high burden of PTB in Uganda, mostly being secondary to HIV [2, 12], the incidence of CPA is probably higher but the index of clinical suspicion for CPA is very low [13]. This may be partly contributed by the shortage of diagnostic capabilities due to lack of local capacity for mycology. Recently, two diagnostic algorithms have been published to facilitate the diagnosis of CPA in resource-constrained settings [14, 15], with emphasis on the exclusion of mycobacterial infections as an important alternative diagnosis in resource-limited settings.

Based on the unified case definition of CPA in resource-limited settings [14], and the point-of-care test, our index of clinical suspicion recently increased, and we started identifying more cases of CPA in our pulmonology wards at Kiruddu National Referral Hospital in Kampala which were previously clinically misdiagnosed and managed as PTB [16, 17]. Herein, we report three cases of CPA that were previously misdiagnosed as PTB in routine clinical practice in a resource-limited setting, using a simple clinical algorithm and the recently validated *Aspergillus* LFD (LDBio Diagnostic, Lyon, France) [18]. This article was written following the CARE Checklist of information to include when writing a case report (Additional file 1).

## Case presentations

### Case I

A 45-year-old Ugandan woman presented with a 3-month history of coughing up blood-stained sputum associated with left-sided pleuritic chest pain, exacerbated by lying on the same side. This was the first episode of hemoptysis. She reported mild dizziness, but no exertional intolerance, orthopnea or ankle swellings. She had no history of constitutional symptoms. She was a peasant farmer who resided in a rural area and was referred from a peripheral health facility where she had been suspected to have TB and was started on anti-TB therapy for 14 days. She was HIV negative and had no history of previous chronic chest symptoms. She was not a current or ex-smoker of tobacco.

On examination, her blood pressure was 120/80 mmHg, pulse rate 80 beats per minute (bpm), peripheral oxygen saturation (SpO_2_) 98% on ambient air, respiratory rate 24 bpm and temperature 36.8 °C. She had no conjunctivae pallor, jaundice or lymphadenopathy. On chest examination, she was tachypneic with mild respiratory distress. She had reduced chest movements on the left with increased tactile fremitus, dull percussion notes and bronchial breathing in the left infraclavicular region. The trachea was deviated to left with the apex beat displaced to the sixth intercostal space (ICS). There were coarse crepitations on the left axillary region. The remainder of the examinations was unremarkable.

**Fig. 1:**
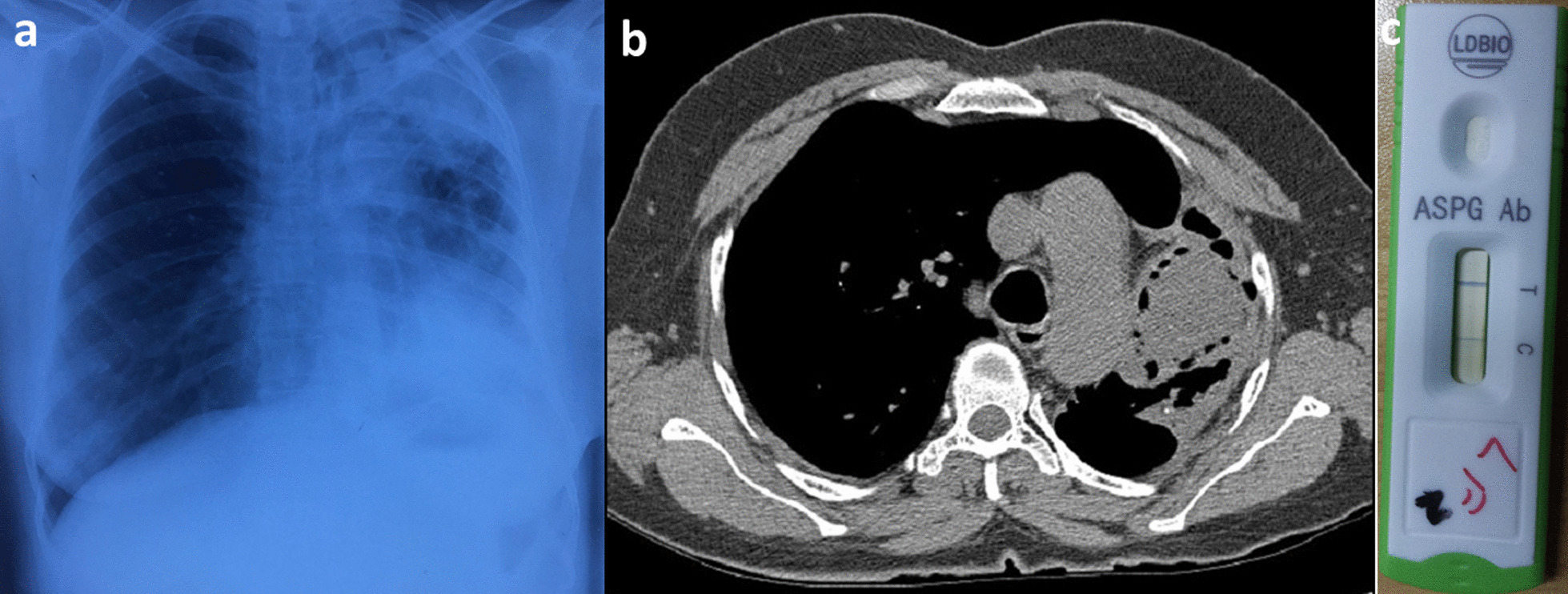
Case 1. **a** Chest x-ray showing a mass in the left upper lung zones with an “air crescent sign” and fibrotic changes in the lower zones. **b** Chest CT scan showing an ovoid mass surrounded by pockets of air, most probably a fungal ball with local pleural thickening. **c** Positive *Aspergillus* IgM-IgG LFD

Chest x-ray showed an intracavitary mass crowned with cresenteric lucency in the left upper zones; the lower zones were dense with fibrotic bands with an associated mediastinal shift to the ipsilateral side and contralateral lung hyperinflation (Fig. 1a). Chest computed tomography (CT) scan showed a cavitating lesion containing a soft tissue density mass with massive architectural distortion. The pleura appeared thickened, and there was herniation of the contralateral lung to the left due to volume loss (Fig. 1b).

The complete blood count was normal. Sputum GeneXpert was negative for *Mycobacterium tuberculosis*. Renal and liver function tests were within normal limits. *Aspergillus*-specific LFD (immunoglobulin (Ig)G/IgM) was positive (Fig. 1c). She had a normal resting electrocardiogram, and echocardiography was of a heart with normal function and structure. A diagnosis of chronic fibrosing and cavitary aspergillosis was made. Anti-TB drugs were stopped, and oral itraconazole 200 mg twice daily was initiated for a minimum duration of 6 months. The patient is alive and doing well and still attends the pulmonology clinic. We had planned to do a control x-ray a few weeks later, but the patient feared traveling back because of the outbreak of the COVID-19 pandemic at that time.

### Case II

A 53-year-old Ugandan woman with a long-standing history of cough presented with a worsening cough over the past 2 weeks. Her cough was productive of a copious brownish foul-smelling sputum that was non-blood-stained associated with right pleuritic chest pain, low-grade fevers, anorexia, marked weight loss and excessive night sweats. She also reported difficulty in breathing on exertion and on lying flat, but showed no lower limb swelling. She was a food vendor in a taxi park who resided in an urban area and had been diagnosed with HIV infection 11 years previously and had been on dolutegravir/lamivudine/tenofovir (DTG/3TC/TDF) for 4 months. Recent CD4 T-counts and viral loads were not known. We did not perform CD4 at that time point. Review of other systems was essentially normal. She was diagnosed and treated four times (6 months each time) for PTB. She had a history of frequent childhood respiratory symptoms of cough and difficulty in breathing, and she was told she suffered from asthma. However, she had no previous documentation and had never used inhaler therapies. She had previously smoke the local tobacco for 2 years and had occasionally used alcohol for 1 year.

On examination, she had a BP of 120/80 mmHg, pulse rate 80 bpm (regular), respiratory rate 22 bpm and SpO_2_ 98% on room air. She was wasted with mild pallor of the mucous membrane with digital clubbing. She had no jaundice, edema or lymphadenopathy. On chest examinations, she had mild respiratory distress. She had a flattened right-side chest in the infraclavicular region with reduced chest movements, tracheal deviation to the right and apex beat of the sixth intercostal space (ICS). In the same chest region, there was increased tactile fremitus, a dull percussion note, bronchial breathing and increased vocal resonance. Cardiac and abdominal examinations were normal.

**Fig. 2:**
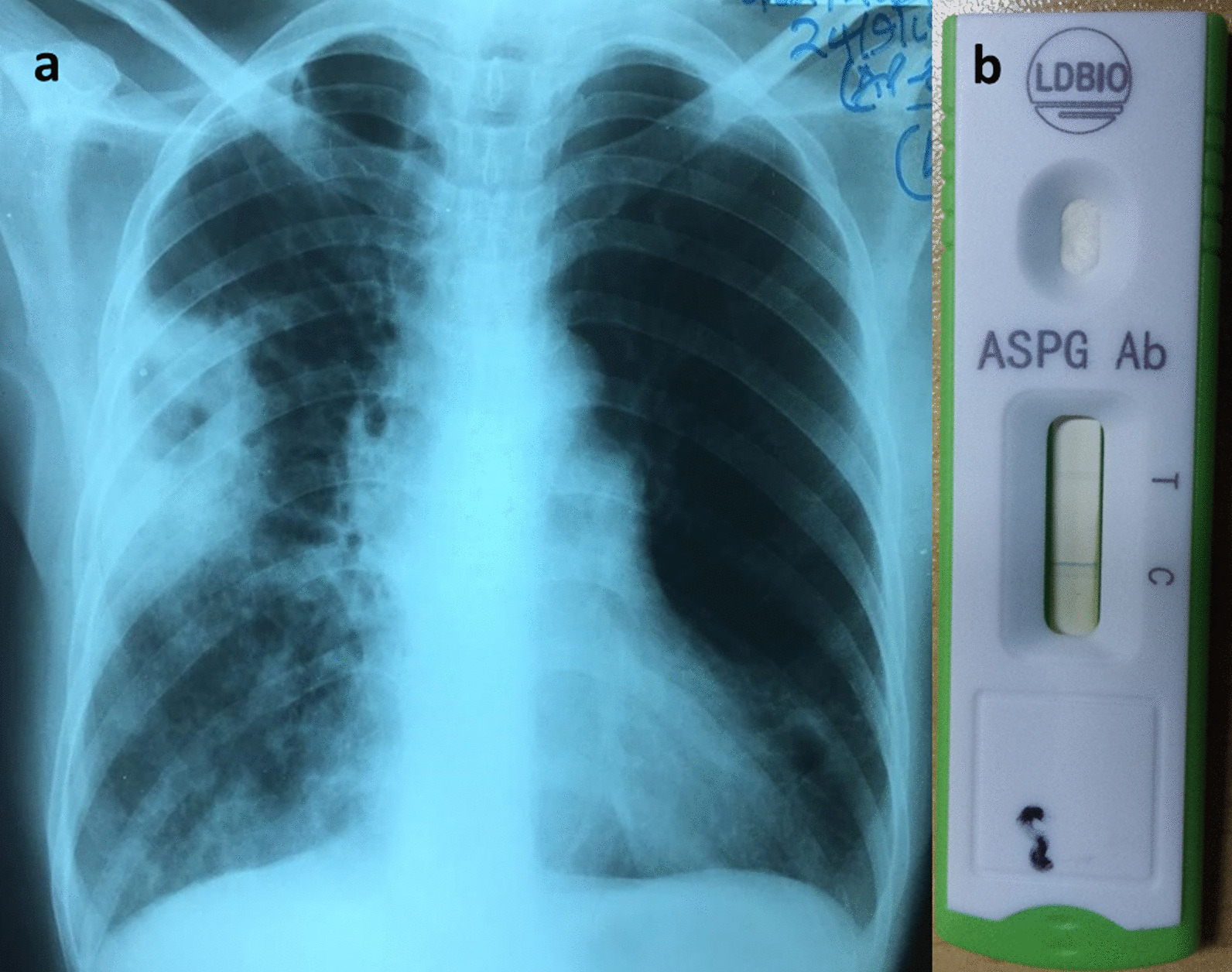
Case 2. **a** Chest x-ray showing a thick-walled cavity with an intrinsic mass in the right middle zone surrounded with patchy opacities medially and inferiorly. **b** Positive *Aspergillus* IgM-IgG LFD

Chest x-ray showed a thick walled cavity with irregular margins containing an intracavitary mass in the right middle zones with perilesional patchy opacities (Fig. 2a). Chest CT scan was not done for this patient. All other laboratory findings were normal apart from the positive *Aspergillus* IgM-IgG LFD (Fig. 2b). The patient was started on oral itraconazole 200 mg twice a day. The patient later died in another hospital.

### Case III

An 18-year-old Ugandan woman, HIV negative, presented with a 2-month history of progressive productive cough, non-blood-stained sputum associated with right-sided pleuritic chest pain exacerbated on coughing and occasional difficulty breathing. She also had associated high-grade fevers, anorexia and marked weight loss but no excessive night sweats. She had no orthopnea or paroxysmal nocturnal dyspnea or lower limb swelling. She was a student who resided in a rural area and was treated for PTB two times in 2017 and 2018 with completion of treatment (6 months for each time).

On examination, she had a blood pressure of 110/70 mmHg, respiratory rate 22 breaths per minute, pulse rate 131 beats per minute and SpO_2_ 94% on room air. She was febrile with a temperature of 38.3 ºC. She was severely pale with no digital clubbing. She had no palpable lymph nodes and no edema. Chest examination revealed a flattened right infraclavicular region with tracheal deviation to the right without displacement of the point of maximum cardiac intensity. There were reduced chest movements on the right hemithorax, with an increased tactile fremitus, dull percussion note and broncho-breathing both posteriorly in the mid-scapular area and in the anterior mammary area. Cardiac examination revealed tachycardia with normal heart sounds.

**Fig. 3:**
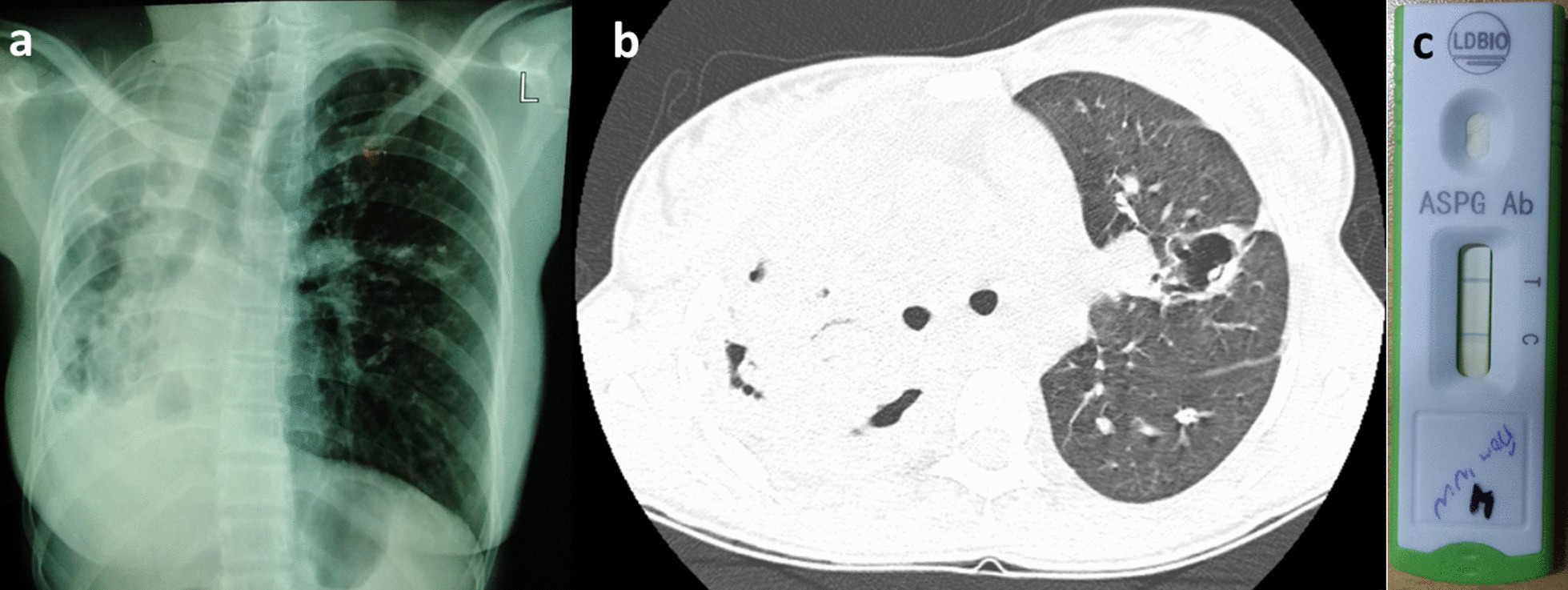
Case 3. **a** Chest x-ray showing post-inflammatory fibrotic changes in the right lung with features of bronchiectasis on the left with a left perihilar cavity. **b** Chest CT scan showing a thick-walled cavity with an intracavitary nodule and bronchiectasis. **c** Positive *Aspergillus* IgM-IgG LFD

Chest x-ray showed a homogeneous opacity in the right hemithorax with multiple crowded cysts in the middle and lower lung zones associated with volume loss and ipsilateral mediastinal shift. There was a cavity in the right perihilar region. There were multiple reticulo-nodular opacities scattered throughout the entire right lung field with cystic changes (Fig. 3a). The chest CT scan showed a thick-walled cavity with an intracavitary nodule in the lingula segment in the lung window. The mediastinal window had a soft tissue density in the right lung field with intrinsic dilated bronchioles. The mediastinum was deviated to the right (Fig. 3b).

Complete blood count showed a leukocytosis, neutrophilia and microcytic anemia. Sputum GeneXpert was negative for *Mycobacterium tuberculosis*. Renal and liver function tests were within normal limits. *Aspergillus*-specific LFD (IgG-IgM) was positive (Fig. 3c). Diagnosis of chronic fibrosing pulmonary aspergillosis with a superimposed bacteremia was made. Oral itraconazole 200 mg twice a day was initiated together with intravenous ceftriaxone 1 g and levofloxacin 500 mg once a day. She was also transfused with 1 unit of whole blood. She developed superior vena cava obstruction (SVCO) and Horner’s syndrome from an aneurysm. We referred her to another private referral hospital for respiratory support and cardiothoracic surgery where she later died.

## Discussion and conclusion

According to the unified case definition of CPA in resource-limited settings [14], CPA can be defined as “illness of ≥ 3 months and all of: (1) weight loss, persistent cough and/or haemoptysis; (2) chest images showing progressive cavitary infiltrates and/or a fungal ball and/or pericavitary fibrosis or infiltrates or pleural thickening; and (3) a positive *Aspergillus* IgG assay or other evidence of *Aspergillus* infection.” A recent publication from our setting [17] showed that we could diagnose CPA in routine clinical practice in a resource-limited setting using a lateral flow device [19–21] as the third criterion (evidence of *Aspergillus* infection) after exclusion of an alternative diagnosis. The LDBio *Aspergillus* LFD has a sensitivity of 92% and a specificity of 98.0% in patients with proven CPA [18]. In this study, patients I and III represent typical cases of CPA. Patient I was initiated on anti-TB medication based on clinical suspicion without microbiological evidence of PTB. Previous studies have shown that almost 35% of subjects with CPA might be wrongly treated with anti-TB medication despite the lack of microbiological evidence [22]. This is also very common in resource-limited settings where the index of clinical suspicion for fungal infections is very low [13, 23] and the diagnostic capacity for mycoses is lacking. A recent paper from our setting showed that “using freely available online materials on medical mycology can enhance teaching and learning of medical mycology" [24].

Patients II and III were typical of post-TB lung disease. This was an expected observation in these patients since persistence of pulmonary cavities after successful pulmonary TB treatment is very common [4] and these cavities are thought to harbor mold spores leading to fungal colonization [11]. So, since patient II was diagnosed and treated four times for PTB, it is possible that not all of these four times were confirmed PTB but were managed as smear-negative or culture-negative TB based on the high clinical suspicion of TB. Moreover, case II appears to be semi-invasive aspergillosis.

CPA is heavily underdiagnosed and misdiagnosed in resource-limited settings where rapid diagnostics are unavailable [25]. Previously treated TB is the most common risk factor for the development of CPA even in the developed world [4, 26]. The burden of CPA attributed to healed TB lesions alone has been estimated at 3347 cases in Uganda [8]. In high tuberculosis burden nations, exclusion of active tuberculosis is the most important first step in the diagnosis of CPA. This is often not an issue, as highly sensitive molecular point-of-care tests, such as the GeneXpert MTB/RIF cartridge-based nucleic acid amplification test, are widely available in Uganda [27]. However, the true burden of post-TB CPA in resource-limited areas endemic for both HIV and TB is not well described, and therefore more epidemiological studies are needed.

A well-designed cross-sectional study conducted in a resource-limited setting (Lagos, Nigeria) with over 70% of the participants being HIV positive reported an overall CPA prevalence of 8.7%, 6.5% among HIV infected and 14.5% among HIV-negative participants [25]. Another study conducted in Gulu, Uganda, reported an overall CPA prevalence of 4.9%, 3% among HIV infected and 6.9% among HIV-negative persons [28]. From these two studies, it appears that CPA may be less frequent in HIV co-infected patients. However, more studies are required to make a concrete conclusion.

As illustrated in the above cases, we have seen patients being subjected to anti-TB medications two or more times even when the sputum test for TB was negative. In addition, obvious cavitary lesions with fungal balls consistent with CPA are treated as active TB lesions because CPA cannot be ruled in. This could be due to the low index of suspicion among clinicians and/or lack of point-of-care fungal diagnostics. We have been able to safely discontinue anti-TBs in patients with negative mycobacterial work-ups who meet the diagnostic criteria for CPA and have commenced them on the appropriate antifungal therapy with an overall good outcome. However, the high cost of itraconazole together with the long duration of treatment is still a major challenge in our country.

Based on the experience and the overwhelming success of LFDs in the diagnosis of a sister fungal infection, cryptococcal meningitis, in resource-limited settings (29, 30), if made widely available and accessible, *Aspergillus* LFD will change the way CPA is diagnosed in resource-limited settings, reducing misdiagnosis and unnecessary use of anti-TB therapy in this population. There is need to advocate for translational research into developing more fungal diagnostics to address the local disease burden [31]. In conclusion, the availability of simple clinical diagnostic criteria for CPA and an LFD have the potential to reduce misdiagnosis of CPA and in turn improve CPA treatment outcomes in resource-limited settings.

## Supplementary Information

**Additional file 1: CARE Checklist.** File contains a CARE Checklist of information to include when writing a case report.

## Data Availability

All data generated or analyzed during this study are included in this published article and its supplementary information files. The authors confirm that all data underlying the findings are fully available without restriction and can be accessed by contacting Mr. Richard Kwizera (kwizerarichard@ymail.com).

## Abbreviations

CPA: Chronic pulmonary aspergillosis
LFD: Lateral flow device
IgG: Immunoglobulin G
PTB: Pulmonary tuberculosis
TB: Tuberculosis
HIV: Human immunodeficiency virus
IgM: Immunoglobulin M
WHO: World Health Organization
COPD: Chronic obstructive pulmonary disease
ICS: Intercostal space
CT: Computed tomography
TDF/3TC/DTG: Tenofovir disoproxil fumarate/ lamivudine/ dolutegravir
CD4: Cluster of differentiation 4
BP: Blood pressure
SPO2: Peripheral capillary oxygen saturation
mmHg: Millimetre of mercury
MTB/RIF: Mycobacteria tuberculosis/rifampicin resistance
CE: Conformité Européene
ICT: Immuno-chromatographic test
BALF: Bronchoalveolar lavage fluid
