# Supplementary material for: Misdiagnosis of chronic pulmonary aspergillosis as pulmonary tuberculosis at a tertiary care center in Uganda: a case series
Source: J Med Case Rep. 2021 Mar 30;15:140. doi: 10.1186/s13256-021-02721-9 (PMC8007227; doi:10.1186/s13256-021-02721-9)
Supplement: Supplementary file 1 — Additional file 1: CARE Checklist. File contains a CARE Checklist of information to include when writing a case report. [file 13256_2021_2721_MOESM1_ESM.docx]

**CARE Checklist of information to include when writing a case report**

| **Item** | **Topic** | **Checklist item description** | **Line/page** |
| --- | --- | --- | --- |
| 1 | **Title** | The words “case report” should be in the title along with the area of focus | **1-2/1** |
| 2 | **Keywords** | Four to seven key words—include “case report” as one of the key words | **48-50/3** |
| 3a | **Abstract** | **Background:** What does this case report add to the medical literature? | **23-32/2** |
| 3b |  | **Case Presentation:** chief complaint, diagnoses, interventions, and outcomes | **33-42/2** |
| 3c |  | **Conclusion:** What is the main “take-away” message from this case? | **43-46/2** |
| 4 | **Introduction/background** | The current standard of care and contributions of this case—with references (1-2 paragraphs) | **52-89/3-4** |
| 5 | **Timeline** | Information from this case report organized into a timeline (table or figure) | **NA** |
| 6a | **Patient Information** | De-identified demographic and other patient or client specific information | **93/4**  **124/6**  **150/7** |
| 6b |  | Chief complaint—what prompted this visit? | **93-95/4**  **124-128/6**  **150-153/7** |
| 6c |  | Relevant history including past interventions and outcomes | **93-99/4-5**  **124-134/6**  **150-155/7** |
| 7 | **Physical Exam** | Relevant physical examination findings | **100-107/5**  **135-142/6**  **156-164/7** |
| 8a | **Diagnostic** | Evaluations such as surveys, laboratory testing, imaging, etc. | **108-117/5**  **143-147/7**  **165-174/7-8** |
| 8b | **Assessment** | Diagnostic reasoning including other diagnoses considered and challenges | **117-121/5-6**  **145-147/7**  **174-177/8** |
| 8c |  | Consider tables or figures linking assessment, diagnoses and interventions | **NA** |
| 8d |  | Prognostic characteristics where applicable | **NA** |
| 9a | **Interventions** | Types such as life-style recommendations, treatments, medications, surgery | **118-119/5**  **146-147/7**  **175-177/8** |
| 9b |  | Intervention administration such as dosage, frequency and duration | **118-119/5**  **146-147/7**  **175-177/8** |
| 9c |  | Note changes in intervention with explanation | **NA** |
| 9d |  | Other concurrent interventions | **NA** |
| 10a | **Follow-up and** | Clinician assessment (and patient or client assessed outcomes when appropriate) | **NA** |
| 10b | **Outcomes** | Important follow-up diagnostic evaluations | **NA** |
| 10c |  | Assessment of intervention adherence and tolerability, including adverse events | **NA** |
| 11a | **Discussion** | Strengths and limitations in your approach to this case | **180-183/8** |
| 11b |  | Specify how this case report informs practice or Clinical Practice Guidelines (CPG) | **180-195/8-9** |
| 11c |  | How does this case report suggest a testable hypothesis? | **212-227/9-10** |
| 11d |  | Conclusions and rationale | **221-227/10** |
| 12 | **Patient Perspective** | When appropriate include the assessment of the patient or client on this episode of care | **NA** |
| 13 | **Informed Consent** | Informed consent from the person who is the subject of this case report | **253-255/11** |
| 14 | **Additional Information** | Acknowledgement section; Competing Interests (Conflict of Interests statement); IRB approval, (Ethical Committee Approval) when required | **238-261/11** |

**Summary of the case**

| 1 | **Patient (gender, age)** | F/45, F/53, F/18 |
| --- | --- | --- |
| 2 | **Final Diagnosis** | Chronic pulmonary aspergillosis |
| 3 | **Symptoms** | longstanding history of cough, chest pain, weight lost and constitutional symptoms |
| 4 | **Medications** | Itraconazole 200mg twice daily for at least 6 months |
| 5 | **Clinical Procedure** | Chest examination, chest radiology, blood investigations |
| 6 | **Specialty** | Mycology |
